# Europium-Induced Ferromagnetism on Bismuth Germanium Oxide Nanoparticles toward Spintronics Applications

**DOI:** 10.1021/acsomega.4c06795

**Published:** 2025-03-18

**Authors:** C. Belman-Rodriguez, J. Guerrero-Sánchez, J. López-Medina, Subhash Sharma, Naji Tabaray, C. Velez, A. Reyes-Serrato, Mario H. Farías, Sergio A. Aguila, R. Ponce-Perez

**Affiliations:** † Centro de Nanociencias y Nanotecnología, Universidad Nacional Autónoma de México, AP 14, Ensenada, Baja California 22860, Mexico; ‡ SECIHTI - IxM - Centro de Nanociencias y Nanotecnología, UNAM. Km 107 Carretera Tijuana-Ensenada s/n. B.C., Ensenada C.P.22800, Mexico; § Department of Electrical Engineering and computer science, University of California, Irvine, California 90095, United States; ∥ Department of Mechanical and Aerospace Engineering, University of California, Irvine, California 90095, United States

## Abstract

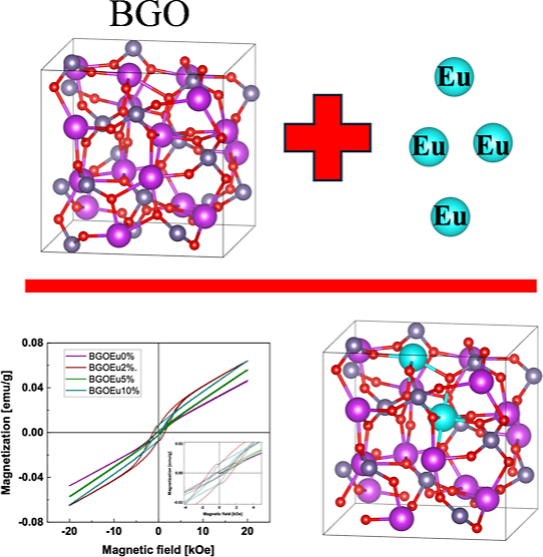

Phosphors of Bi_4_Ge_3_O_12_:Eu^3+^ were obtained by using the polymeric precursor route. After confirming the Eu incorporated into the Bi_4_Ge_3_O_12_ lattice, we analyzed the magnetic behavior in the systems with 0%, 2%, 5%, and 10%. The magnetization vs magnetic field measurements evidenced ferromagnetism in all the samples, with different magnetization saturation values. In the pristine structure, the magnetic behavior is directly related to Bi vacancies, where the neighbor O atoms induce spin polarization. The ferromagnetic characteristics are enhanced in the 2% Eu content because Eu atoms take on the Bi vacancy sites. In contrast, the ferromagnetic character diminishes as the Eu content is increased to 5%, which is directly correlated with site competence as Eu can occupy either Bi or Ge sites. Upon raising the Eu percentage to 10%, the ferromagnetic character gets strengthened. In this case, we hypothesize that zones of the material with a high concentration of Eu may appear with ferromagnetic alignment, confirmed by our ab initio computations. The evidence generated in this paper lays the foundations for considering rare-earth-doped Bi_4_Ge_3_O_12_ for applications in the spintronics industry.

## 1 Introduction

Bismuth germanium oxide (Bi_4_Ge_3_O_12_), from now on BGO, has a cubic Eulytine-like structure with a space group (*I*43̅*d*, 220). It possesses distorted BiO_6_ octahedrons connected to GeO_4_ tetrahedrons. In this structure, Bi and Ge behave as Bi^3+^ and Ge^4+^, respectively, while oxygen is called O^2–^. The electronegativity of the species is as follows: Ge(2.01) < Bi(2.02) < O (3.44). When doping this structure, the entering species may accommodate in either site depending on their electronegativity with their neighbors.

Doping is a way to engineer material properties to improve their versatility and reach a wider range of applications. Depending on the species of impurities, BGO may host them in different sites.^1,2^ If the electronegativity and the atomic radius are similar, then it is expected to have atom-by-atom exchanges in which the impurity removes one atom of the matrix. Species may replace oxygen with large electronegativities, while for electronegativities reached, either Bi or Ge can be replaced.

Several studies have appeared in the literature in which doping is treated. For example, Mg- and Ca-doped BGO single crystals exhibit a near-infrared emission with the emission center related to Bi atoms.^3^ Due to the large difference in electronegativities between Mg and Ca with O, Bi or Ge may be replaced by Mg or Ca. Still, evidence is needed to determine which site is most favorable. Doping with transition metals like W has also been reported.^4^ The W-doped BGO exhibits an emission intensity stronger than pristine BGO under ultraviolet radiation that is even improved after thermal treatment under a N_2_ atmosphere.^4^

Rare earth ions are interesting for doping BGO since they also possess attractive optical properties from ultraviolet to infrared.^5^ There is evidence that BGO doped with several lanthanides enhances the radiation resistance and excessive light output.^6^ In contrast to bare BGO, Ce-doped BGO possesses a high radiation resistance under ultraviolet radiation due to a reduced concentration of color centers generated by electron or hole traps, faster fluorescence lifetime, and 30% larger luminescence intensity.^6^ Pr-doped BGO exhibits luminescence and near-infrared emission due to Bi and sharp-emission Pr peaks.^7,8^ Furthermore, Eu-doped BGO evidences high thermal stability and enhanced photoluminescence,^9^ useful for white light emission^9^ devices and visible light lasers.^10^

Other rare earths have been employed to engineer BGO toward new applications. For example, Dy-doped BGO phosphor is an efficient solid-state lighting device,^11^ and when doped with Er, may be an optical temperature sensor^12^ and a scintillation detector since it presents luminescence in the near-infrared range.^13^ Yb-doped BGO also presents near-infrared emission,^14^ making it useful for similar applications.

When entering into the BGO matrix, all rare earth impurities evidence a 3^+^ ionization state, almost assuring that they take on the Bi sites in the octahedrons, generating certain distortions that may induce the different properties and emission ranges. Recently, evidence has appeared that rare earth may not only be in the Bi octahedron but can also be located in the Ge tetrahedral sites.^15^ Both structures are stable, and notably, they confer bifunctionality when selectively doping BGO. Quantum mechanical calculations predicted that the Bi site induces ferromagnetism (FM) and the Ge site, antiferromagnetism (AFM).^15^ However, empirical measurements are required to confirm that, indeed, magnetism is induced due to rare earths in the BGO matrix.

In this work, we follow up the theoretical and experimental assessment of Eu-doped BGO as a model system to demonstrate the emergence of magnetism due to rare earths. We anticipate that weak ferromagnetic characteristics were measured and depend on the Eu doping concentration. As the Eu concentration increases, a competence between ferromagnetism and antiferromagnetism appears, mediated by Eu replacing Bi and Ge sites simultaneously, attenuating the ferromagnetism in the samples. Our results are a step forward in considering the magnetic behavior induced by rare earths in the multifunctional properties observed in doped BGO.

## 2 Methods

### 2.1 Experimental Procedure

Synthesis of the samples occurred via a polymeric precursor route. Bismuth nitrate (Bi(NO_3_)_3_ 98.0%, Sigma-Aldrich), germanium oxide (GeO_2_, 99.999% Alfa Aesar), and europium nitrate (Eu(NO_3_)_3_, 99.9%, Sigma-Aldrich) served as precursors, while l(+)-tartaric acid (C_4_H_6_O_6_, 99.5% Sigma-Aldrich) acted as the chelating agent.

### 2.2 Synthesis Conditions

Phosphors of Bi_4_Ge_3_O_12_/Eu^3+^ were obtained using the polymeric precursor route. All starting materials were of analytical-grade quality, following the procedure outlined by Y. Chu et al.,^9^ and no additional purification was carried out. The europium molar ratio was systematically varied at 2.0%, 5.0%, and 10.0% molar of Eu^3+^. For comparison, an undoped BGO sample was also synthesized. The precursor oxides and nitrates were combined in stoichiometric proportions. GeO_2_, Bi(NO_3_)_3_, and Eu(NO_3_)_3_ were dissolved in 50 mL of 30% HNO_3_, and the mixture was stirred for 2 h at room temperature. Subsequently, tartaric acid was dissolved in 15 mL of deionized water, mixed with the precursors, and stirred for 24 h. The resulting mixture was heated to 80 °C for 2 h, and the sol was further heated to 100 °C until dry, forming a xerogel. Finally, the xerogel underwent annealing at 850 °C for 4 h with a heating rate of 2 °C/min.

### 2.3 X-ray Diffraction (XRD)

The powders were characterized by using X-ray diffraction (XRD). Diffraction patterns were obtained with a Phillips X’pert diffractometer, utilizing Cu Kα radiation (λ = 0.15406 nm). Measurements were conducted over a 2θ range of 10–80°, with a step size of 0.02° and a dwell time of 0.5 s per point.

### 2.4 Scanning Electron Microscopy

Scanning electron microscopy (SEM) images were acquired using a JEOL JSM 5300 at a magnification of 3000×. For SEM analysis, each sample was mounted on carbon tape prior to imaging.

### 2.5 Computational Procedure

The Eu-doped BGO systems were modeled according to the density functional theory (DFT) framework implemented in the Vienna Ab-initio Simulation Package (VASP) code.^16,17^ The exchange–correlation energy was modeled using the generalized gradient approximation (GGA) with the Perdew–Burke–Ernzerhof (PBE) parametrization.^18^ The electron–ion interactions are threaded by the projector Augmented-Waves (PAW) pseudopotentials^19,20^ with 450 eV as the energy cutoff. Since Eu has highly correlated f-electrons, the Hubbard correction (DFT + U) is employed according to the simplified (rotationally invariant) approach introduced by Dudarev et al.^21^ with *U*_eff_ = 4 eV as in previous reports.^15^ In geometry optimization, convergence is achieved when all the force components and the energy differences are less than 0.01 eV/Å and 1 × 10^–7^ eV, respectively. The Brillouin zone is sampled using the Monkhorst–Pack *k*-point set with a 6 × 6 × 6 grid.

## 3 Results and Discussions

### 3.1 Structural Analysis

**Figure 1:**
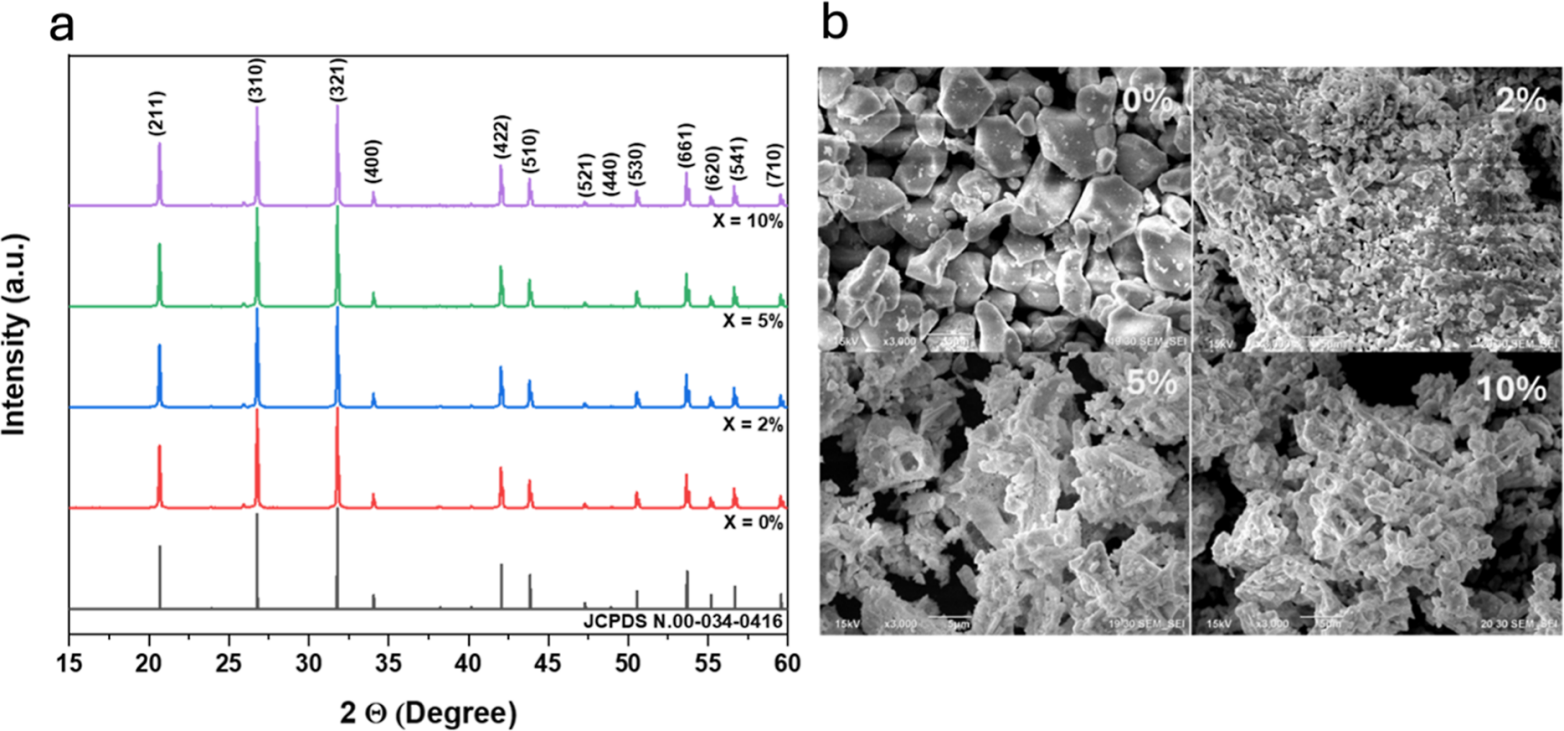
(a) XRD and (b) SEM characterization of BGO doped with different amounts of Eu.

This section analyzes the BGO structure at different Eu concentrations through X-ray diffraction (XRD) and scanning electron microscopy (SEM) measurements. First, we have obtained bare BGO in its cubic phase, as seen in the lower panel in Figure 1a (red line). It is also evident that doping with Eu does not change the XRD patterns, ensuring that Eu is effectively entering the BGO lattice. In Figure 1a, a minor impurity is observed at 27.8° 2θ, which, according to the JCPDS crystallographic card 00-027-0050, corresponds to the (210) plane of Bi_2_O_3_. This impurity is present in all of the samples, including the undoped BGO, and thus its contribution to the magnetic activity of the material is considered negligible. Here, Eu presumably takes on Bi sites and, to a lesser extent, Ge sites.

Based on the XRD data, the lattice parameters were calculated, yielding the following results: for *x* = 0.00, a (Å) = 10.5186; V (Å^3^) = 1163.78, *x* = 0.02, a (Å) = 10.6175; V (Å^3^) = 1196.92, *x* = 0.05, a (Å) = 10.5188; V (Å^3^) = 1163.854, *x* = 0.10, a (Å) = 10.5195; V (Å^3^) = 1164.08. It is observed that as Eu ions are introduced into the BGO lattice, there is a slight increase in the cell volume. Furthermore, the most significant increase in volume occurs when the BGO is doped with 2% Eu.

FTIR analyses were performed (Figure S1), revealing that after the addition of the Eu^3+^ dopant ion, signals at 516 and 585 cm^–1^ appear, corresponding to the vibrational modes of α-Bi_2_O_3_ and the Bi–O bond stretching vibrations, respectively. Meanwhile, signals at 553 cm^–1^, 840 cm^–1^, and 960 cm^–1^, and a broad band centered around 910 cm^–1^ are associated with the formation of GeO_2_. The presence of GeO_2_ may result from a potential segregation process, as Eu^3+^ ions compete for Ge^4+^ sites. These measurements suggest that in certain regions of the crystalline lattice, Eu^3+^ can displace Ge^4+^. However, it is important to highlight that the formation of GeO_2_ occurs on the material surface and in small quantities. As such, this GeO_2_ formation is undetectable by XRD.^15^

The upper left panel in Figure 1b shows nanostructured BGO. As Eu enters the BGO lattice and its content increases, the nanoparticle size gets reduced; see the upper right and bottom panels from Figure 1b; this is a common phenomenon that occurs when the doping species are cations and are being integrated into the lattice.

### 3.2 Magnetic Analysis

**Figure 2:**
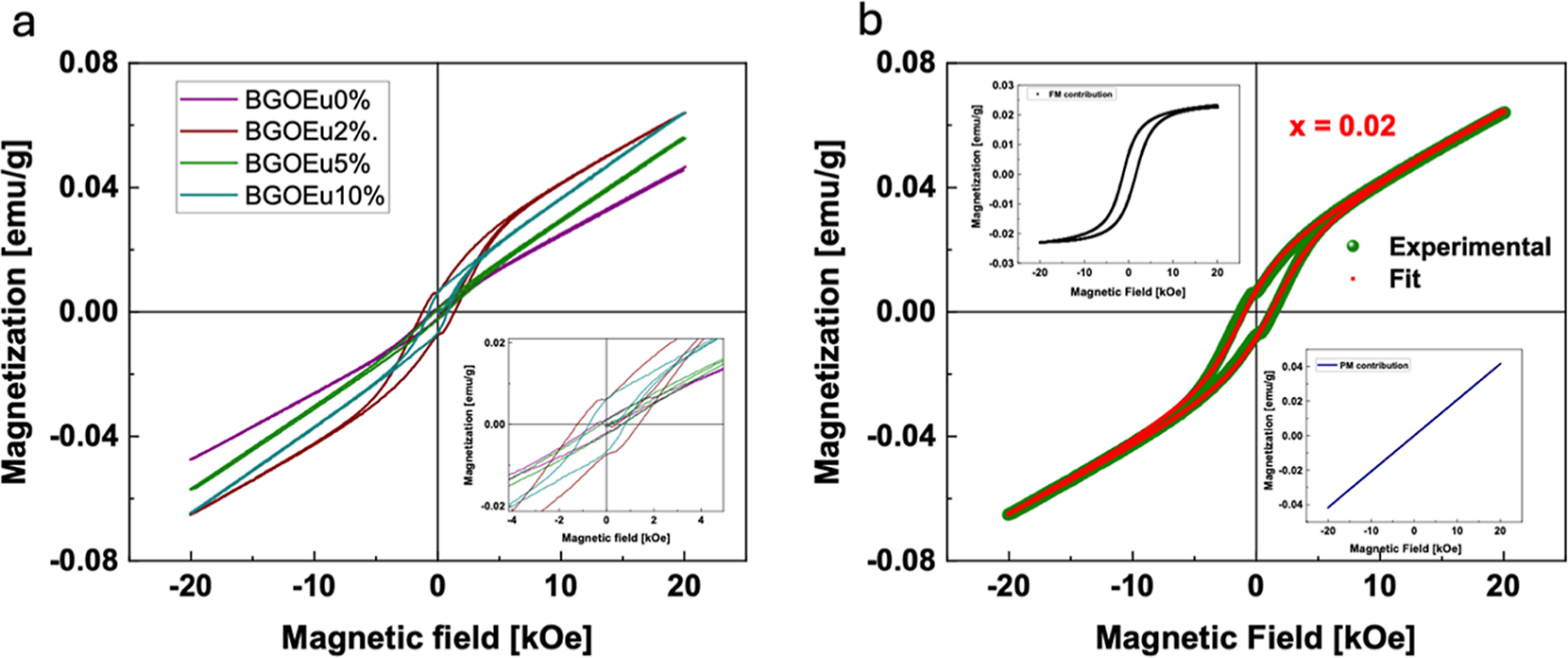
(a) The M–H curves of BGO: Eu^3+^ at room temperature. (b) Deconvoluted M–H loop for BGO with *x* = 0.02.

Once we determined that the BGO crystal structure remains unchanged by the presence of Eu, we proceeded to determine the magnetic response of the Eu-doped materials. The magnetization (*M*) vs magnetic field (*H*) measurements were carried out at room temperature in the range of 20 kOe. The *M* vs *H* curves are shown in Figure 2a. It is noted that the weak ferromagnetic ordering is observed for *x* = 0.0 (5.17 × 10^–5^ emu/g), which increases with Eu doping in the BGO network and reaches 19.0 × 10^–5^ emu/g for *x* = 0.10. It is observed that Eu doping modified the magnetic properties of BGO samples. It is worth mentioning here that the M_r_ increases with Eu doping and reaches a maximum for *x* = 0.02 (21.2 × 10^–5^ emu/g); after that, it decreases for *x* = 0.05 (4.27 × 10^–5^ emu/g), and again it increases for *x* = 0.10 (19.0 × 10^–5^ emu/g). The coercive field (*H*_c_) values increase with Eu doping, as seen in Table 1. In such cases, a weak ferromagnetic ordering is present in the samples. It is important to extract the FM and AFM contributions from the data. So, we fitted the M–H curves with the following equation^22,23^
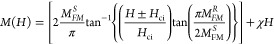
1In this equation, χ denotes the magnetic susceptibility of the antiferromagnetic and/or paramagnetic (PM) part. Further, in the first term,  is the ferromagnetic saturation magnetization,  denotes the remnant magnetization, and *H*_ci_ is the intrinsic coercivity. Figure 2b shows the typical fitted curve for *x* = 0.02, and the upper inset shows the FM contribution, whereas the lower inset shows the AFM contribution. The same approach was used for the other samples. The fitting magnetic parameters are listed in Table 1. The remnant magnetization has the same trend as the experimental data. The deconvolution magnetic data confirm the FM ordering in these samples. This behavior can be explained based on Eu possibility to take either Bi or Ge sites, which is further proved by DFT calculations in the next section.

**Table 1:** Magnetic Parameter of All Samples in BGO: Eu^3+^ of M–H Curves Measured at Room Temperature for the FM, AFM, and PM Contributions

% Eu	magnetic fitting parameters				experimental parameters	
	M_r_ (10^–3^) (emu/g)	M_S_(10^–3^)(emu/g)	H_C_ (Oe)	χ(10^–6^)	M_r_ (10^–5^) (emu/g)	H_C_ (Oe)
*x* = 0.0	1.24	5.18	1177	2.0812	5.17	577
*x* = 0.02	6.84	12.27	1426	2.0877	21.2	1293
*x* = 0.05	1.37	8.00	1417	2.3780	4.27	360
*x* = 0.10	5.50	9.50	1105	2.7439	19.0	780

### 3.3 Quantum Mechanical Calculations

In our previous work,^15^ we predicted the appearance of FM in BGO when Eu takes on Bi sites and AFM when Eu replaces Ge atoms.^15^ In the previous section, we have compelling experimental evidence that the BGO is magnetic. However, there is an interesting behavior in the sample, in which at *x* = 0.0, the material evidences weak ferromagnetism probably induced by vacancies; when increasing the Eu concentration to *x* = 0.02, magnetism increases, but when reaching *x* = 0.05, the ferromagnetic character gets attenuated, and finally for *x* = 0.10, the magnetic response gets recovered. To analyze such behavior, we performed quantum mechanical calculations to describe the appearance of magnetism due to vacancies in BGO and the possibility of site competence when Eu enters the lattice at a large concentration, leading to competence between ferromagnetism and antiferromagnetism.

Vacancies stability and its role in the magnetic properties of BGO

**Figure 3:**
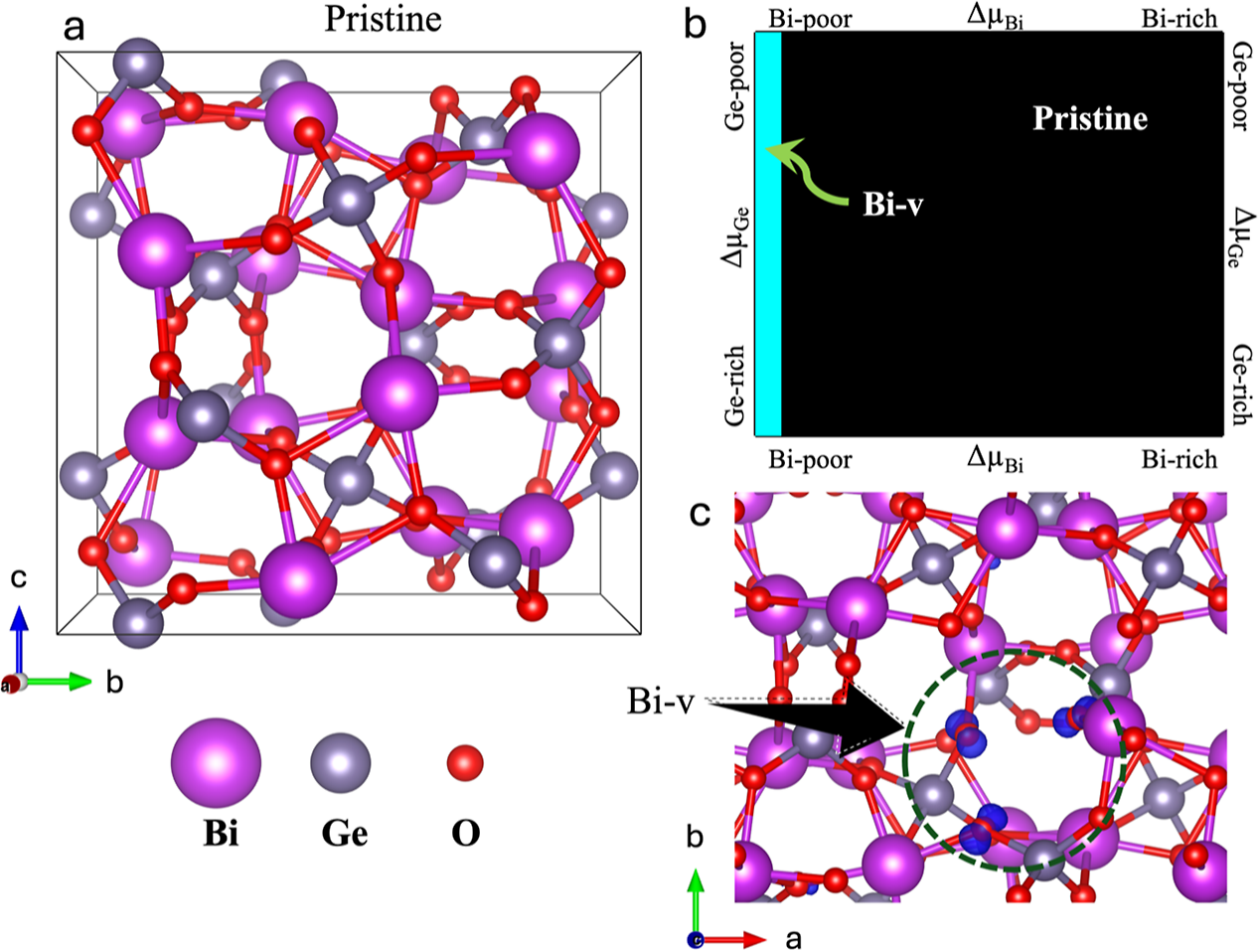
(a) Bi_4_Ge_3_O_12_ pristine atomic structure. (b) Vacancy formation energy phase diagram Δμ_Bi_ vs Δμ_Ge_ (blue and black planes stand for Bi vacancies and pristine BGO). (c) Bi vacancy in the BGO structure; in blue are depicted the p-orbital-like shape spin-density isosurfaces around the O atoms nearby the vacancy site. Eu-induced magnetism in BGO.

To analyze the possibility of magnetism induced by vacancies in BGO (see the BGO crystal structure in Figure 3a), we studied the appearance of such intrinsic defects and their effect on the electronic and magnetic properties. The stability of three vacancies, Bi, Ge, and O, was analyzed through Defect Formation Energies^24^ since total energies are not a parameter to compare when different numbers of atoms are treated in the models. The DFE can be written as follows
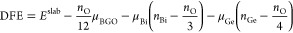
2

where *E*^slab^ is the total energy of the system at hand and *n*_i_ (μ_i_) is the number of atoms (chemical potential) of the *i*th specie. Figure 3b depicts the vacancy formation energy phase diagram under different Bi and Ge growth conditions. In the case of Bi, the Δμ_Bi_ goes from Bi-rich (μ_Bi_ = μ_Bi_^bulk^) to Bi-poor () conditions, and in the case of Ge from Ge-rich (μ_Ge_ = μ_Bi_^bulk^) to Ge-poor () conditions. -ΔH is the heat of enthalpy of formation of the BGO compound, which allows us to map almost all possibilities that may appear at different Ge and Bi contents. Also, the growth conditions at the O-rich condition appear for the Bi-poor and Ge-poor conditions. Notice from the figure that at almost all growth conditions, the pristine structures are the most stable; however, for Bi-poor and at all Ge growth ranges, the Bi vacancies are stable (cyan color in Figure 3b). Ge and O vacancies are less stable than Bi vacancies, which are intrinsic, and they will always appear in BGO. Our findings agree with Zyryanov et al.^25^ By XRD measurement and structure refinement, they find that Bi- and Ge-v are present in the structure (Bi_3.39_Bi0.092+Ge_3_O_11.4_). Also, they suggest that the Bi vacancies result from placing a Bi atom in a tetrahedral site. The charge compensation is carried out by the reduction of some Bi ions or by the formation of Bi vacancies

To proceed with the analysis of vacancy-induced magnetism in the BGO, we proceed to plot the spin-density isosurfaces of the most stable vacancy model. Figure 3c shows that the Bi vacancy generates a p-orbital-like shape spin isosurface to the neighboring O atoms due to the unsaturated bonds. Each oxygen atom provides an induced magnetization of 0.27 μ_B_. There are also vacancy-induced atomic rearrangements that are needed to reach the local stability. Considering the shape and size of the spin densities, we expect a weak ferromagnetic character induced by the vacancies. These findings agree fully with our experimental observations.

Once we know that BGO presents a weak ferromagnetic character due to Bi vacancies, we will analyze the role of Eu in the electronic and magnetic properties of BGO as another source of magnetism. When Eu is incorporated into the BGO lattice, we must analyze the competence among Bi, Ge, or mixed sites. For the lowest percentage (*x* = 0.02), the experimental evidence points to a ferromagnetic character, as seen in Figure 2a. In such a case, we expect that Eu will primarily occupy the Bi vacancy sites; then the O dangling bonds will be saturated, and the magnetic response will be enhanced due to f-orbital-induced magnetism. In fact, the Eu taking on Bi sites is highly stable for a large chemical potential region,^15^ so the Bi vacancies are highly favorable sites for Eu incorporation.

When increasing the Eu percentage to *x* = 0.05, the system experiences a magnetism attenuation, probably due to the competence between AFM and FM alignments that appear when Eu also takes on Ge sites. Remember that Eu in Bi sites is ferromagnetic when Eu impurities are separated, and the combination of Eu in separated Bi and Ge sites ends up with antiferromagnetic characteristics and is 4 meV more stable than ferromagnetic alignment. With this analysis, we hypothesize that Eu impurities in *x* = 0.05 are well dispersed, showing a large competence between antiferromagnetism and ferromagnetism, which ends up in the attenuation of the total magnetic moments and then to a weak ferromagnetic behavior.

**Figure 4:**
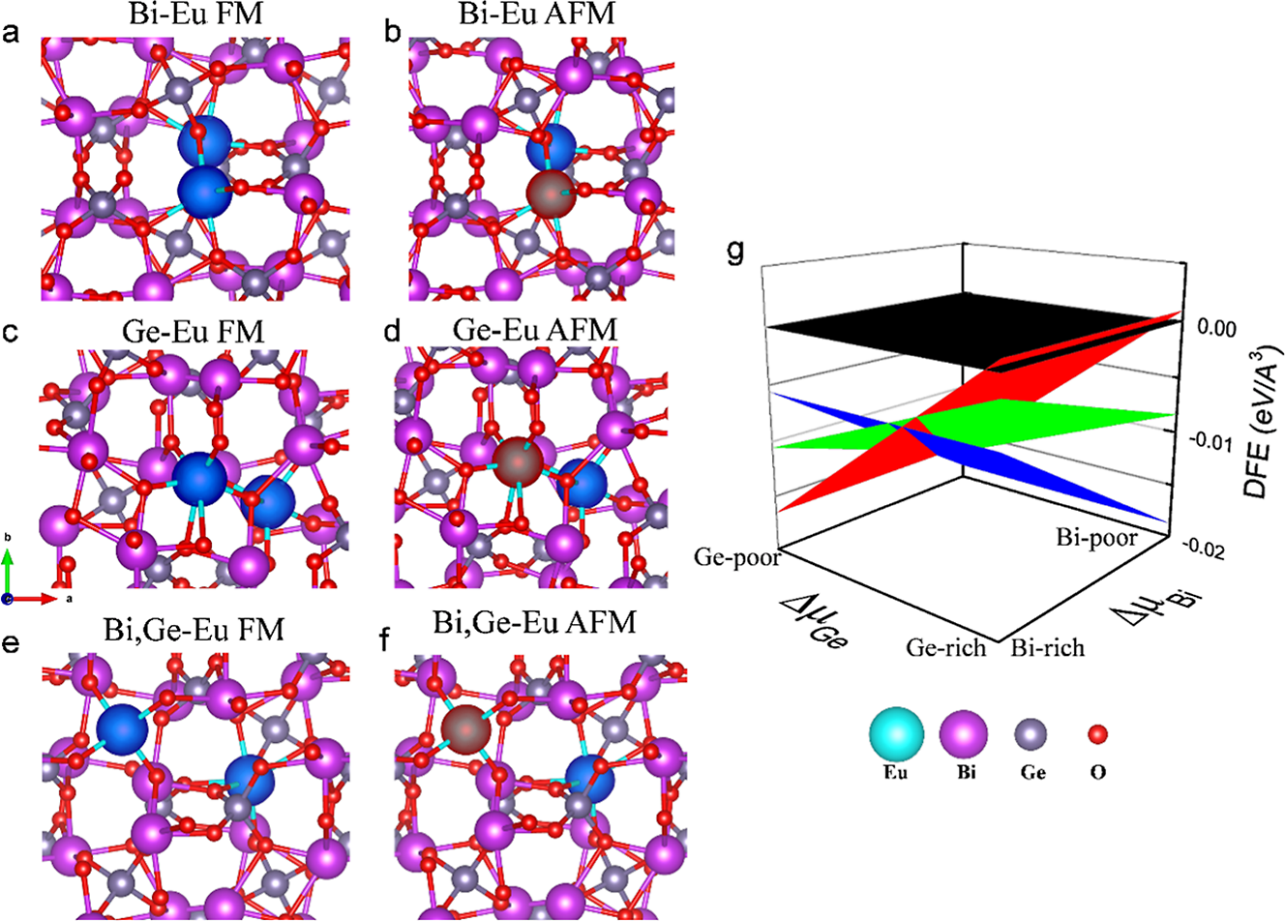
Atomistic models for two Eu atoms at the (a) Bi site in ferromagnetic alignment, (b) Bi site in antiferromagnetic alignment, (c) Ge site in ferromagnetic alignment, and (d) Ge site in antiferromagnetic alignment, (e) Bi and Ge sites with ferromagnetic alignment, and (f) Bi and Ge sites with antiferromagnetic alignment. (g) Defect formation energy plot vs (Δμ_Bi_, Δμ_Ge_).

For the Eu content *x* = 0.1, Bi vacancies get occupied, and some small regions of the host present a high presence of Eu atoms, which may be combinations of sites, for example, Bi–Bi, Ge–Ge, or even Bi–Ge sites (see Figure 4a–f). Figure 4a stands for Eu incorporated in a pair of Bi sites with ferromagnetic alignment, and Figure 4b is the same but considers antiferromagnetic alignment between Eu atoms. Figure 4c,d exemplifies the Eu-occupying Ge sites in ferromagnetic and antiferromagnetic alignments. Finally, Figure 4e,f depicts the atomic models when Eu takes on Bi and Ge sites simultaneously in ferromagnetic and antiferromagnetic alignments, respectively. We calculated all these potential candidates and analyzed their stability vs the Ge and Bi chemical potentials using the following equation.
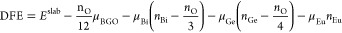
3which is equal to eq 2, but it includes the term μ_Eu_*n*_Eu_ that accounts for the changes in DFE energy by incorporating Eu atoms in either Bi or Ge vacancies or by replacing Bi or Ge sites. Here, μ_Eu_ is the chemical potential of the Eu bulk structure and n_Eu_ is the number of Eu atoms added to the system.

Figure 4g depicts the defect formation energy as a function of Δμ_Bi_ and Δμ_Ge_. The red plane stands for the Eu taking on Ge sites, blue is for Eu occupying Bi sites, green is for a combination of Eu placed in Bi and Ge sites, and black stands for pristine BGO. Notice that when incorporating pairs of Eu atoms, the systems become more stable (provide lower energy values) than the pristine structure (black plane), so there is more probability of finding Eu pairs or even regions of the host material with a high Eu content, in which FM dominates. We also compared the energetics of antiferromagnetic and ferromagnetic alignments in these sites. In the Bi–Bi site, the ferromagnetic alignment between Eu atoms is 0.22 meV more stable than the antiferromagnetic alignment; when Eu takes one Ge–Ge site, the difference in energy between FM and AFM is 2.66 meV, with the ferromagnetic alignment being favored. Finally, when Eu takes on Bi and Ge sites, the AFM alignment is 6 meV more stable than the ferromagnetic alignment. Figure 4a–f depicts the spin density isosurfaces of the Eu pair at different sites and with different magnetic alignments.

### 3.4 Electronic Properties

**Figure 5:**
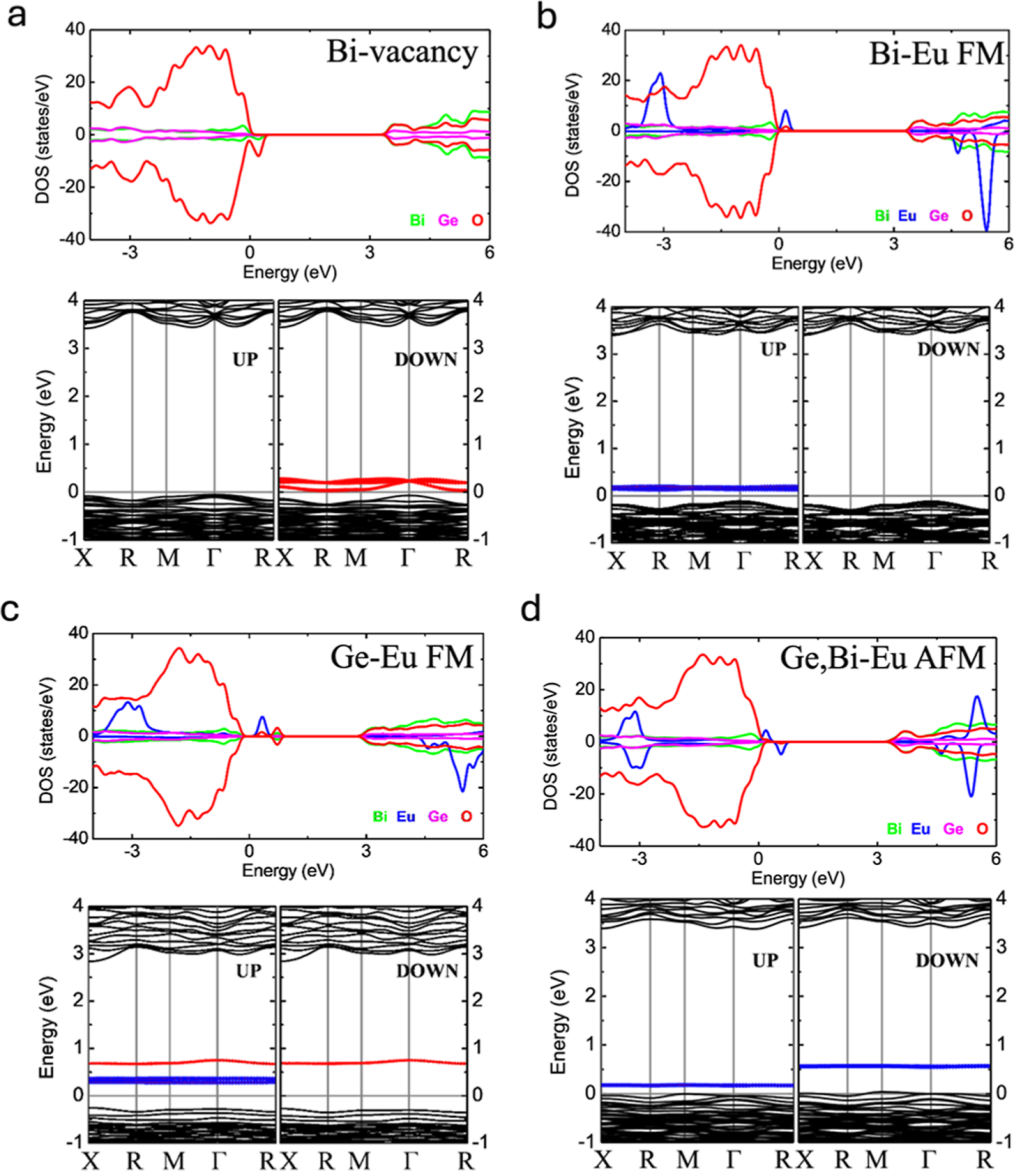
DOS and band plot diagram for (a) Bi vacancy model, (b) Eu incorporated into the Bi–Bi sites, (c) Eu incorporated into the Ge–Ge sites, and (d) Eu incorporated into neighbor Bi–Ge sites. Fermi energy is set to zero.

In this section, we describe the electronic properties of the most stable structures, Bi vacancy, Eu incorporated in Bi–Bi sites, Eu incorporated in Ge–Ge sites, and, finally, Eu incorporated in the Bi–Ge sites, in all Eu pairs. In all configurations, we focus on the case where the Eu atoms are close to each other, since it is the most favorable configuration. Electronic characterization is carried out by calculating the projected density of states (DOS) and band plot diagrams along the X-R-*M*-Γ-R path. In all cases, the energy reference is the Fermi level; besides, positive (negative) values along the DOS axis in the density of states graphs are for spin up (down). Figure 5a depicts the DOS and band plot diagram for the Bi vacancy model; notice that the ferromagnetic character in the structure is mainly induced by the asymmetry generated by O atoms near the Fermi energy, and at the unoccupied states, magnetism is induced by acceptor impurities that polarize spin-down. In the case of Eu incorporated into Bi–Bi sites (Figure 5b) each Eu has a magnetic moment of 6.20 μ_B_. It is noticed that Eu atoms introduce states at lower and higher energies far from the Fermi energy and that these states hybridize with the O atoms. Also, there appears to be a peak attributed to Eu atoms around the Fermi energy as an unoccupied state, which also hybridizes with the O atoms. These contributions induce FM character in this structure. The other atoms do not participate with a magnetic character. Notice also that the Eu states near the Fermi energy are acceptor states, which also modulate the band gap in the majority spin channel.

Now we analyze the Eu incorporated into the Ge–Ge sites, Figure 5c, where Eu has magnetic moments of 6.24 and 6.17 μ_B_. As in the case of Eu in the Bi–Bi sites, similar characteristics appear at lower and higher energies in the DOS and band structure. However, we have noticed certain changes around the Fermi energy. Although the Eu–O hybridization appears, there is another contribution of the O atoms at around 0.6 eV, which hybridize with Bi atoms. This effect is directly induced by the different oxidation state of the Eu impurities (Eu^3+^) that takes on Ge^4+^ sites in the tetrahedron, weakening the Eu–O bonds while strengthening the Bi–O bonds in its neighborhood. Finally, in Figure 5d, we depict the DOS and band diagram of the Eu ions incorporated into mixed Bi and Ge neighbor sites with Eu magnetic moments of 6.19 and −6.16 μ_B_, respectively. Here, the main interaction is antiferromagnetic as evidenced by the DOS and band diagram, in which although the Eu states around the Fermi energy do not lay at the same energy, they are spin-up/spin-down states and eliminate each other. At lower and higher energies, the AFM alignment induced by the Eu atoms and its hybridization with the O atoms is clear. Notice here that O impurity states do not appear because both sites Bi and Ge are present, stabilizing the electronic imbalance induced by Eu in Ge sites. It is a general trend that the band gap is reduced upon incorporating Eu; these electronic modifications may also induce interesting changes in the BGO emission. Such a study is beyond the scope of the present work. However, our results are a step further in considering the role of magnetism in this kind of rare-earth materials.

## 4 Conclusions

In this work, we have demonstrated the appearance of weak FM in Eu-doped Bi_4_Ge_3_O_12_ nanoparticles through a comprehensive experimental and theoretical assessment. Results have demonstrated that Bi vacancies produce a weak ferromagnetic behavior in Bi_4_Ge_3_O_12_, where the vacancies of the O atoms near the Bi vacancy magnetize due to the unsaturated bonds. When 2% of Eu atoms are introduced into the Bi_4_Ge_3_O_12_ lattice, the ferromagnetic behavior is strengthened because Eu atoms may tend to occupy the Bi vacancy sites. Bi_4_Ge_3_O_12_ has a weak ferromagnetic behavior after increasing the Eu content to 5%. Still, it is attenuated because the competition between Bi (Eu in Bi sites produce FM) and Ge (Eu in Ge sites AFM) sites appears. At 10% Eu, Bi_4_Ge_3_O_12_ also strengthens the ferromagnetic behavior. We hypothesize that at large Eu percentage, some zones are rich in Eu content, and considering that, once more, FM alignments dominate, which is the main reason for the magnetic enhancement. Larger Eu percentages may drive to regions with large Eu content, so the best percentage to generate enhanced FM behavior is 2%. Our study is a step forward in widening the well-known applications in Bi_4_Ge_3_O_12_ toward the spintronics industry.

## Data Availability

Data used are available throughout the manuscript text.

## Supporting Information Available

FTIR of undoped and BGO doped with different concentrations of Eu^3+^ (PDF)
