# Supplementary material for: Europium-Induced Ferromagnetism on Bismuth Germanium Oxide Nanoparticles toward Spintronics Applications
Source: ACS Omega. 2025 Mar 18;10(12):11762–9. doi: 10.1021/acsomega.4c06795 (PMC11966274; doi:10.1021/acsomega.4c06795)
Supplement: Supplementary file 1 — ao4c06795_si_001.pdf [file ao4c06795_si_001.pdf]

Supporting Information for

Europium-induced ferromagnetism on Bismuth Germanium Oxide nanoparticles towards spintronics applications

C. Belman-Rodriguez[a*], J. Guerrero-Sánchez[a*], J. López-Medina[b], Subhash Sharma[b], Naji Tabaray[c], C. Velez[d], A. Reyes-Serrato[a], Mario H. Farías[a], Sergio A. Aguila[a], R. Ponce-Perez[a]

[a] *Centro de Nanociencias y Nanotecnología, Universidad Nacional Autónoma de México, AP 14, Ensenada, Baja California, México, 22860.*

[b] *CONAHCYT - IxM - Centro de Nanociencias y Nanotecnología, UNAM. Km 107 Carretera Tijuana-Ensenada s/n. B.C., C.P. 22800, México.*

[c]*Department of electrical Engineering and computer science, University of California, Irvine, California, USA*

[d]*Department of Mechanical and Aerospace Engineering, University of California, Irvine, California, USA*

e-mail: belman25@ens.cnyn.unam.mx; guerrero@ens.cnyn.unam.mx.

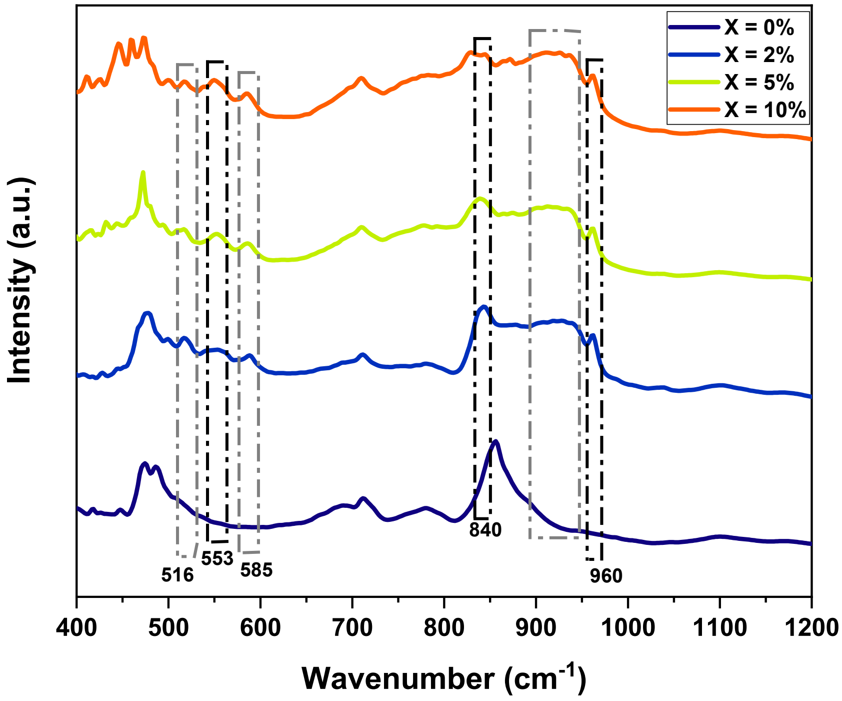

Figure S1. FTIR spectra of undoped BGO and BGO doped with different concentrations of Eu^{3+}.
